# Serum vitamin D levels and Sjogren’s syndrome: bi-directional Mendelian randomization analysis

**DOI:** 10.1186/s13075-023-03062-2

**Published:** 2023-05-15

**Authors:** Meng Zhao, Feiran Wei, Han Li, Zemin Wang, Shuai Wang, Yangyang Liu, Gaoqiang Fei, You Ge, Pingmin Wei

**Affiliations:** 1 Department of Epidemiology and Health Statistics, School of Public Health, Southeast University, Nanjing, 210009 Jiangsu China; 2 Division of Rheumatology, Zhongda Hospital Southeast University, Nanjing, 210008 Jiangsu China

**Keywords:** Mendelian randomization, Vitamin D, 25(OH)D, Sjogren’s syndrome

## Abstract

**Background:**

Based on the results of existing observational studies, it can be found that the association between serum vitamin D levels and the risk of Sjogren’s syndrome (SS) in humans is still controversial. Based on this situation, this study aimed to assess the causal relationship between serum vitamin D levels and SS by using the Mendelian randomization (MR) approach.

**Methods:**

In this study, genome-wide association studies (GWAS) summary statistics on serum vitamin D levels [sample size = 417,580 (UK Biobank)] and SS [sample size = 416,757 (cases = 2495, controls = 414,262) (FinnGen)] were used. The bi-directional MR analysis was then used to assess possible causal relationships. The major analysis method of MR was performed using inverse-variance weighted (IVW), supplemented by MR-Egger and the weighted median approaches. In addition, sensitivity analyses were used to ensure the stability of the results, including Cochran’s *Q* test, MR-PRESSO, MR-Egger intercept test, and the leave-one-out test.

**Results:**

The MR suggested that no significant causal effects of serum 25(OH)D levels on SS risks were observed [odds ratio (OR) = 0.9824; 95% confidence interval (CI) = 0.7130 to 1.3538; *P* = 0.9137]. Similarly, no evidence supported the causal effects of SS on serum vitamin D levels (*β*: 0.0076, 95% CI: − 0.0031 to 0.0183; *P* = 0.1640).

**Conclusion:**

This study found no obvious evidence that serum vitamin D level is causally associated with SS risks or vice versa. We call for larger sample size studies to further unravel the potential causal relationship and the exact mechanism.

**Supplementary Information:**

The online version contains supplementary material available at 10.1186/s13075-023-03062-2.

## Introduction

Sjogren’s syndrome (SS) is a complex, heterogeneous systemic chronic autoimmune disorder commonly presenting with dry eyes and mouth [1–3]. SS is one of the most common autoimmune diseases with a prevalence of 0.1 to 4.8% in various populations, according to the strict definition of the American-European Consensus Criteria [4–6]. SS can cause damage to almost any organ or system causing a variety of complications, including immune thrombocytopenia, interstitial lung disease, autoimmune hepatitis, and lymphoma to name just a few [7], which places a tremendous financial burden on patients’ families and healthcare services [8]. In addition, SS can cause fatigue, depression, anxiety, and decreased physical performance, which in turn seriously affects the patient’s quality of life [5].

Vitamin D is a nutrient with multiple biological effects and its main form in serum is 25-hydroxyvitamin D [25(OH)D], which plays an important role in immune regulation [9, 10]. Currently, low levels of vitamin D due to lack of sunlight exposure or low dietary intake have been identified as a major risk factor for autoimmune diseases [11]. There have been several observational studies exploring the association between vitamin D and SS risk. Recently, two large cross-sectional studies including 107 and 176 SS patients from Turkey [12] and Europe [13] were conducted. The former study found no difference in vitamin D levels between cases and controls, while the latter reported lower levels of vitamin D levels in patients with SS. In addition, a cohort study demonstrated that vitamin D deficiency is common in SS patients [12], and a meta-analysis based on the observational studies also obtained the same results [14]. Based on the above, there are inconsistent results regarding the association between vitamin D levels and SS. Conclusions about causality cannot be drawn solely from the results in observational designs, possibly because of the limitations contained in the cohort and cross-sectional studies (limited sample size, different races, and other existing confounding factors and bias). Currently, it is uncertain whether the relationship between vitamin D and SS is causal, and whether it operates in one or both directions.

Mendelian randomization (MR) analysis is a useful epidemiological research strategy for assessing causal relationships. With the development and advancement of the Human Genome Project, MR analysis uses genetic variants as instrumental variables (IVs), which minimizes the limitations of observational studies and yields unconfounded information on the causal relationship between exposure and outcome through its specific analytical methods [15, 16]. IVs typically use single nucleotide polymorphisms (SNPs) obtained from genome-wide association studies (GWAS), which are DNA sequence polymorphisms induced by single nucleotide mutation within the genome [17]. According to the principle of independent classification (Mendel’s law of random allocation), genetic variants are randomly assigned during meiosis [18]; thus, they can be considered hereditary randomized controlled trials (RCTs) and may not be affected by residual confounding and reverse causality. Based on this, the study was to examine the causal association between serum vitamin D and SS, using the data from large-scale GWAS with the bi-directional MR design.

## Materials and methods

### Ethics

This study was reported according to STROBE-MR guidelines [19]. Data were collected from public databases. And there is no ethical approval necessary.

### Study design

**Fig. 1:**
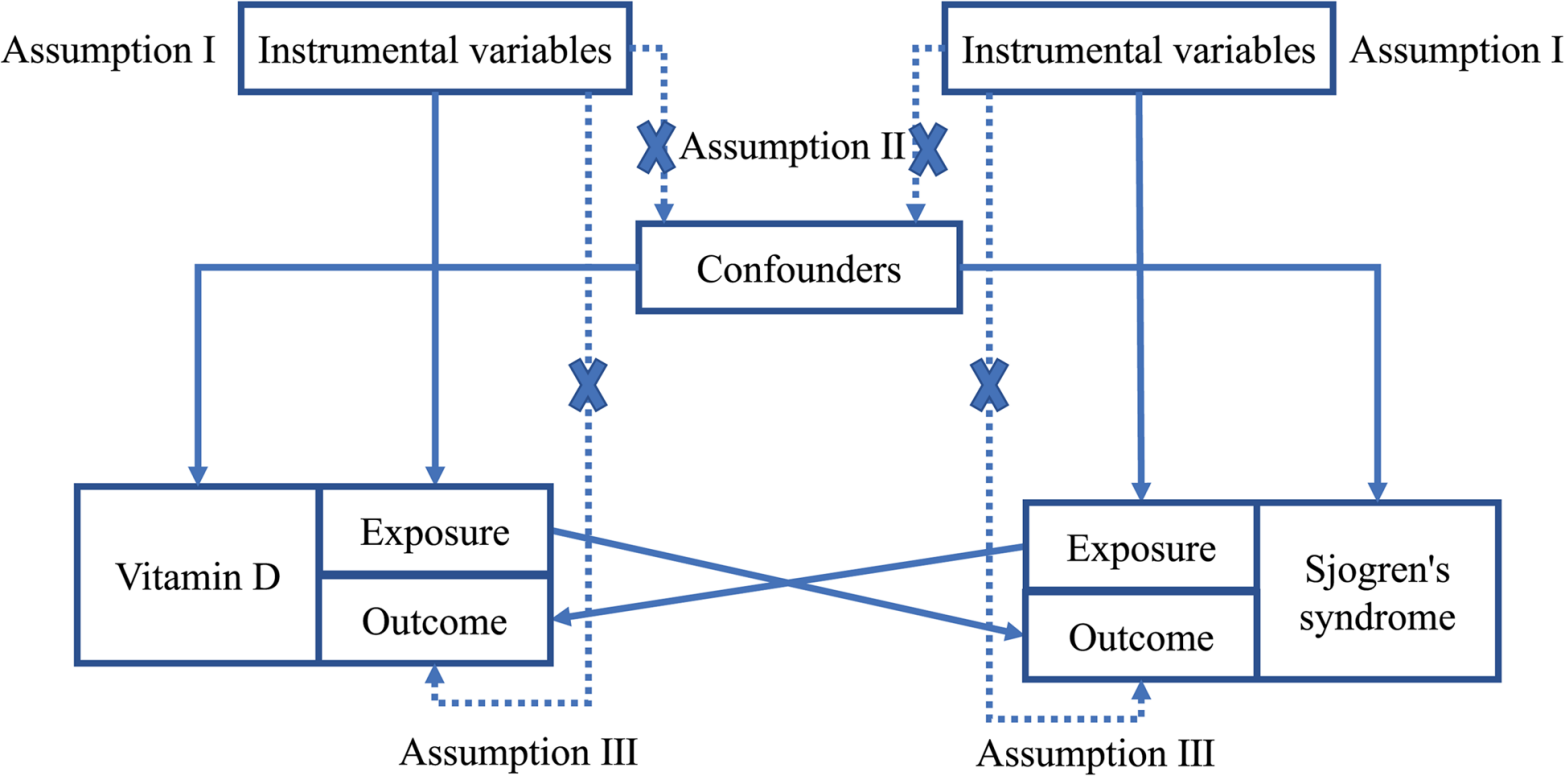
An overview of this MR study design

A bi-directional MR study was conducted to investigate the causal associations between serum vitamin D levels and SS [20]. SNPs are used as IVs for MR analysis to determine the causal effect of exposure variables [21, 22]. It is worth noting that MR analysis is subject to three assumptions [17]: (I) the IVs are closely related to exposure (“relevance”); (II) the IVs are independent of any potential confounding factor (“exchangeability”); (III) the IVs only affect outcome via the exposure (“exclusion restriction”). The framework is described in Fig. 1.

### Data sources for serum 25(OH)D levels and SS

The summary data of serum 25(OH)D levels were obtained from the GWAS study at the UK Biobank [23], including phenotype, genotype, and clinical information on 417,580 individuals of European ancestry (age ranging from 40 to 69 years old) [24]. Serum 25(OH)D levels were quantified by chemiluminescent immunoassay in nanomoles per liter in blood samples collected from 2006 to 2013. Individuals with 25(OH)D levels out of the range of 10–375 nmol/L were excluded. The median, mean, and interquartile range of 25(OH)D levels were 47.9, 49.6, and 33.5 to 63.2 nmol/L, respectively.

Meanwhile, the summary data for SS was available from FinnGen [25] (https://www.finngen.fi/en/) with the phenocode “M13_SJOGREN” which consisted of 416,757 samples (2495 cases; 414,262 controls), and a total of 16,383,308 SNPs were genotyped. The information about each data source is provided in Table 1.

**Table 1:** Description of GWAS used for each phenotype

Phenotype	Data sources	Sample size	SNPs (*n*)	Ancestry
25(OH)D	UK Biobank	417,580	8,806,780	European
SS	FinnGen	416,757 (2495 cases; 414,262 controls)	16,383,308	European

*GWAS* Genome-wide association studies, *25(OH)D* 25-hydroxyvitamin D, *SS* Sjogren’s syndrome, *SNPs*, Single nucleotide polymorphisms

### Selection of genetic variants as IVs

SNPs that were significantly associated with 25(OH)D levels and SS, respectively, were screened from GWAS data as preliminary IVs (*P* < 5 × 10^−8^ at genome-wide threshold). Meanwhile, linkage disequilibrium (LD) analysis was performed to ensure independence between SNPs (LD-r^2^ < 0.001 and clumping distance > 10,000 kb) [17]. Given that the main assumption of the MR analysis is that IVs can only affect the outcome through exposure, we manually eliminated SNPs related to confounders using PhenoScanner [26] (http://phenoscanner.medschl.cam.ac.uk/).

### Testing instrument strength and statistical power

To minimize any possible weak IV bias, the strength of the IV was assessed using the *F*-statistic [27]. We use the following formula $F=\frac{{R}^{2}\times (n-2)}{1-{R}^{2}}$ (*n*: sample size of the GWAS; *R*^2^: the proportion of explained variance of the IV) to calculate *F*-value [28]. A higher *F*-statistic corresponded to a smaller bias [27]. And if *F* > 10, it indicates that the study had sufficient strength [29]. Meanwhile, *R*^2^ was calculated using the formula: ${R}^{2}=2\times {\beta }^{2}\times (1-\mathrm{EAF})\times \mathrm{EAF}$ (*β*: estimate of the genetic effect of each SNP on iron status; EAF: effect allele frequency) [30]. The statistical power of MR analysis was calculated using an online tool at https://shiny.cnsgenomics.com/mRnd/ [31]. Briefly, power is calculated based on the sample size of GWAS, the proportion of cases, and the variance explained by genetic instruments for the exposure.

### MR analysis

Before the MR analysis, the data were harmonized according to the previously described method [20], to correspond to the effect sizes of exposures and outcomes to the same effect alleles. The principal MR analysis was based on inverse-variance weighted (IVW) multiplicative random effects models [20]. The IVW method assumes that the MR assumptions are met or that all SNPs are valid. In addition, MR-Egger [32] and weighted median [33] were used in complementary analyses. MR-Egger analysis was conducted to assess whether the IVs have directional horizontal pleiotropic effects on the outcome [32]. The weighted median method can give a valid causal estimate when more than 50% of the information is derived from valid IVs [33]. However, the power of the weighted median and MR-Egger methods are limited compared to IVW, which tend to provide wider confidence intervals (CI) [20], and are therefore employed in this study only as complementary methods.

### Sensitivity analyses

First, according to Cochran’s *Q* statistic, IV heterogeneity was determined by using the random effects model (*P* < 0.05) or fixed effects model (*P* > 0.05) [34]. Second, horizontal pleiotropy was examined by conducting the MR-Egger intercept test. Meanwhile, horizontal pleiotropy can be detected with the MR-Pleiotropy Residual Sum and Outlier methods (MR-PRESSO) based on both SNP-level and global heterogeneity estimates. To identify outlier variants, the outlier test compares expected and observed distributions of each variant. If any of the outlier variants are detected, they would be discarded to obtain an unbiased causal estimate from an outlier-corrected MR analysis [35]. Third, the leave-one-out method eliminated the included SNPs one by one and calculated the effect of the remaining IVs to evaluate whether the MR estimate was driven or biased by a single SNP, which was performed in the sensitivity analysis [18]. In addition, when vitamin D was used as the exposure and SS was used as the outcome, the measure of the effects was odds ratios (ORs) and its 95% CI; conversely, the effect measure was *β* and its 95% CI.

MR analyses were conducted using R version 4.2.1 with the “TwoSampleMR” [36] (version 0.5.6) and “MR-PRESSO” [37] (version 1.0) R packages. It was considered significant if the two-sided *P*-value was less than 0.05.

## Results

### Characteristics of the selected SNPs

SNPs strongly correlated with 25(OH)D were extracted as IVs in GWAS. The LD analysis was also performed (LD-r^2^ < 0.001, clumping distance > 10,000 kb). Meanwhile, SNPs for risk factors associated with SS (atopic dermatitis [38]: rs1038165, rs10454087, rs12123821, inflammation [39]: rs55814693) were searched (*P* < 1 × 10^−5^) through the PhenoScanner database. In addition, palindromic variants resulting in potential strand ambiguity were removed. Eventually, 81 SNPs were enrolled in the MR analysis of 25(OH)D on SS.

For the IVs of SS, we found that only a small number (*n* = 3) of SNPs were obtained when a strict *P*-value (*P* < 5 × 10^−8^) was taken for screening. To include more SNPs associated with SS, a more lenient threshold was used in this study (*P* < 5 × 10^−7^). After combining LD analysis and searching (*P* < 1 × 10^−5^) the PhenoScanner database for risk factors (basal metabolic rate [40]: rs3093958), four SNPs were finally obtained for the MR analysis of the causal association of SS on 25(OH)D.

The *F*-statistics for IVs were all over 10, suggesting IVs were generally considered to provide sufficient information for MR studies (Supplementary Tables 1 and 2).

### Causal effects of serum 25(OH)D levels on SS risks

**Fig. 2:**
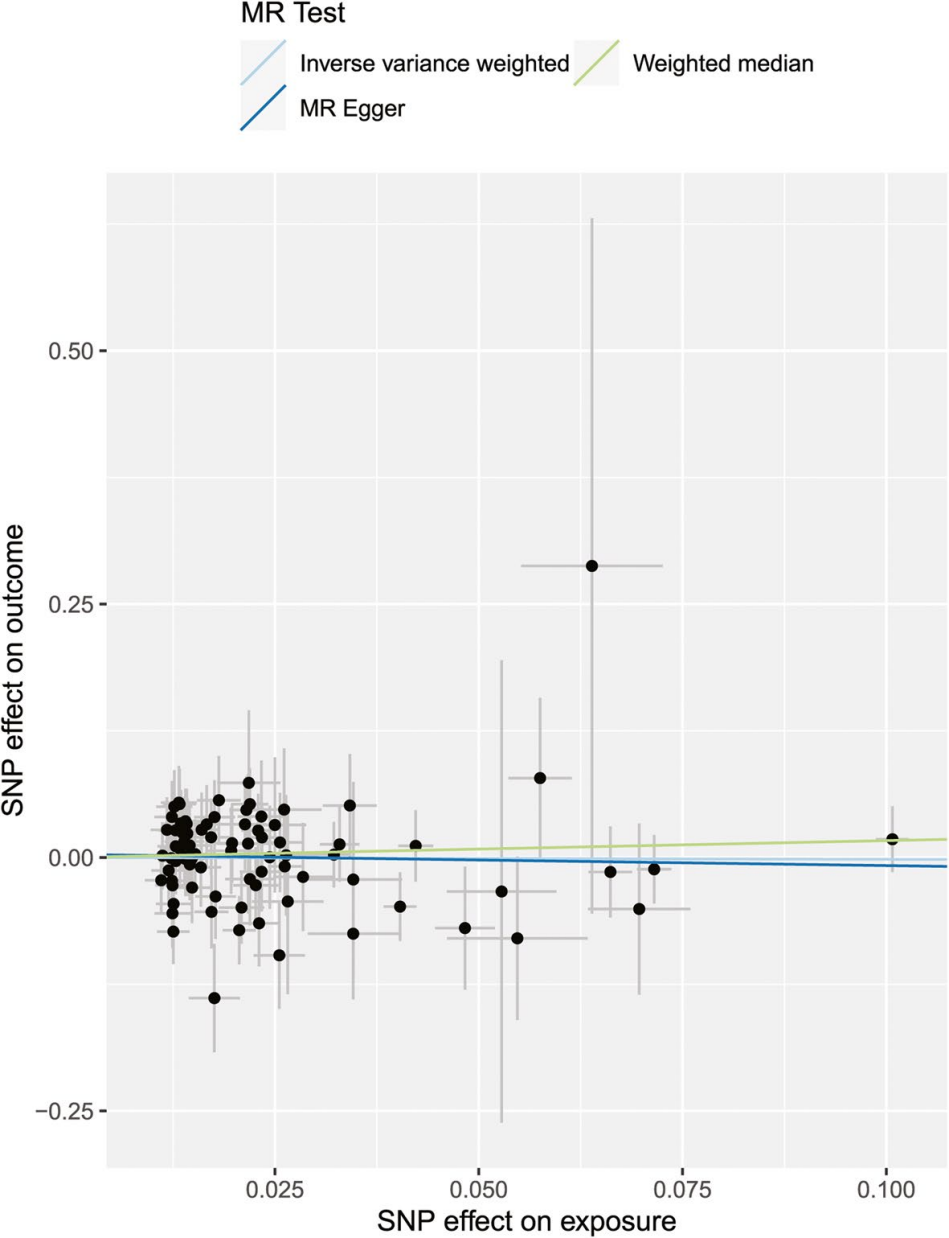
Scatter plot of the causal effect of serum 25(OH)D levels on SS

The results of this MR analysis are shown in Table 2 and Fig. 2, with a power of 0.32. The OR of serum 25(OH)D associated with SS for each of the three methods (IVW, weighted median, and MR-Egger) were 0.9824 (95% CI: 0.7130 to 1.3538, *P* = 0.9137), 1.1814 (95% CI: 0.7054 to 1.9786; *P* = 0.5263), and 0.8955 (95% CI: 0.5209 to 1.5394; *P* = 0.6908). Our results of the three methods all showed that genetically predicted levels of serum 25(OH)D were not significantly associated to the risk of SS (all *P* > 0.05).

**Table 2:** Causal effects of serum 25(OH)D levels on SS risks in MR analysis

Exposure	Outcome	SNPs (*n*)	MR method	OR	95% CI	*P*-value
25(OH)D	SS	81	IVW	0.9824	(0.7130, 1.3538)	0.9137
			Weighted median	1.1814	(0.7054, 1.9786)	0.5263
			MR-Egger (*P* for heterogeneity = 0.7221; *P* for pleiotropy = 0.2787)	0.8955	(0.5209, 1.5394)	0.6908
			MR-PRESSO global test	-	-	0.7483

*25(OH)D*, 25-hydroxyvitamin D, *SS* Sjogren’s syndrome, *SNPs* Single nucleotide polymorphisms, *OR* Odds ratio, *CI* Confidence intervals, *IVW* Inverse-variance weighted

### Causal effects of SS on serum 25(OH)D levels

**Fig. 3:**
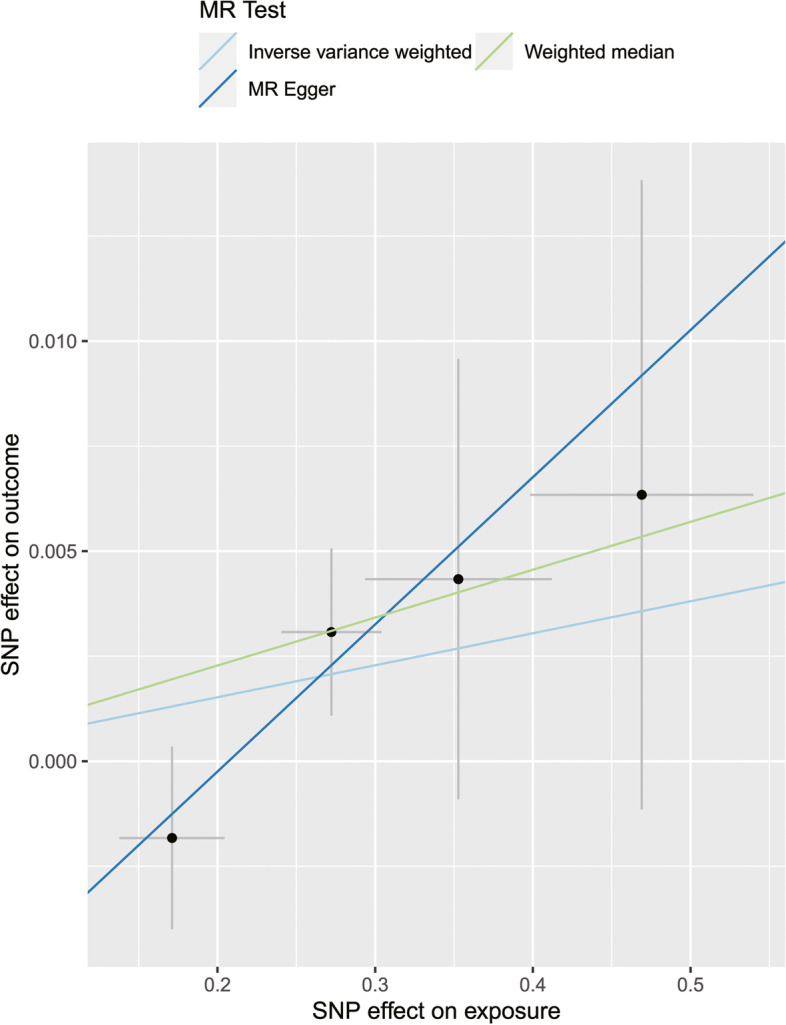
Scatter plot of the causal effect of SS on serum 25(OH)D levels

In Table 3 and Fig. 3, reverse MR results were presented with a low statistical power of 0.11. The *β* of SS associated with serum 25(OH)D for each of the three methods (IVW, weighted median, and MR-Egger) were 0.0076 (95% CI: − 0.0031 to 0.0183; *P* = 0.1640), 0.0114 (95% CI: − 0.0011 to 0.0239; *P* = 0.0773), and 0.0350 (95% CI: − 0.0030 to 0.0731; *P* = 0.2128). The results of the three methods all showed that genetically predicted SS were not significantly associated to levels of serum 25(OH)D (all *P* > 0.05).

**Table 3:** Causal effects of SS on serum 25(OH)D levels in MR analysis

Exposure	Outcome	SNPs (*n*)	MR method	*β*	95% CI	*P*-value
SS	25(OH)D	4	IVW	0.0076	(− 0.0031, 0.0183)	0.1640
			Weighted median	0.0114	(− 0.0011, 0.0239)	0.0773
			MR-Egger (*P* for heterogeneity = 0.8218; *P* for pleiotropy = 0.679)	0.0350	(− 0.0030, 0.0731)	0.2128
			MR-PRESSO global test	-	-	0.5317

*SS* Sjogren’s syndrome, *25(OH)D* 25-hydroxyvitamin D, *SNPs* Single nucleotide polymorphisms, *CI* Confidence intervals, *IVW* Inverse-variance weighted

### Sensitivity analyses of MR

**Fig. 4:**
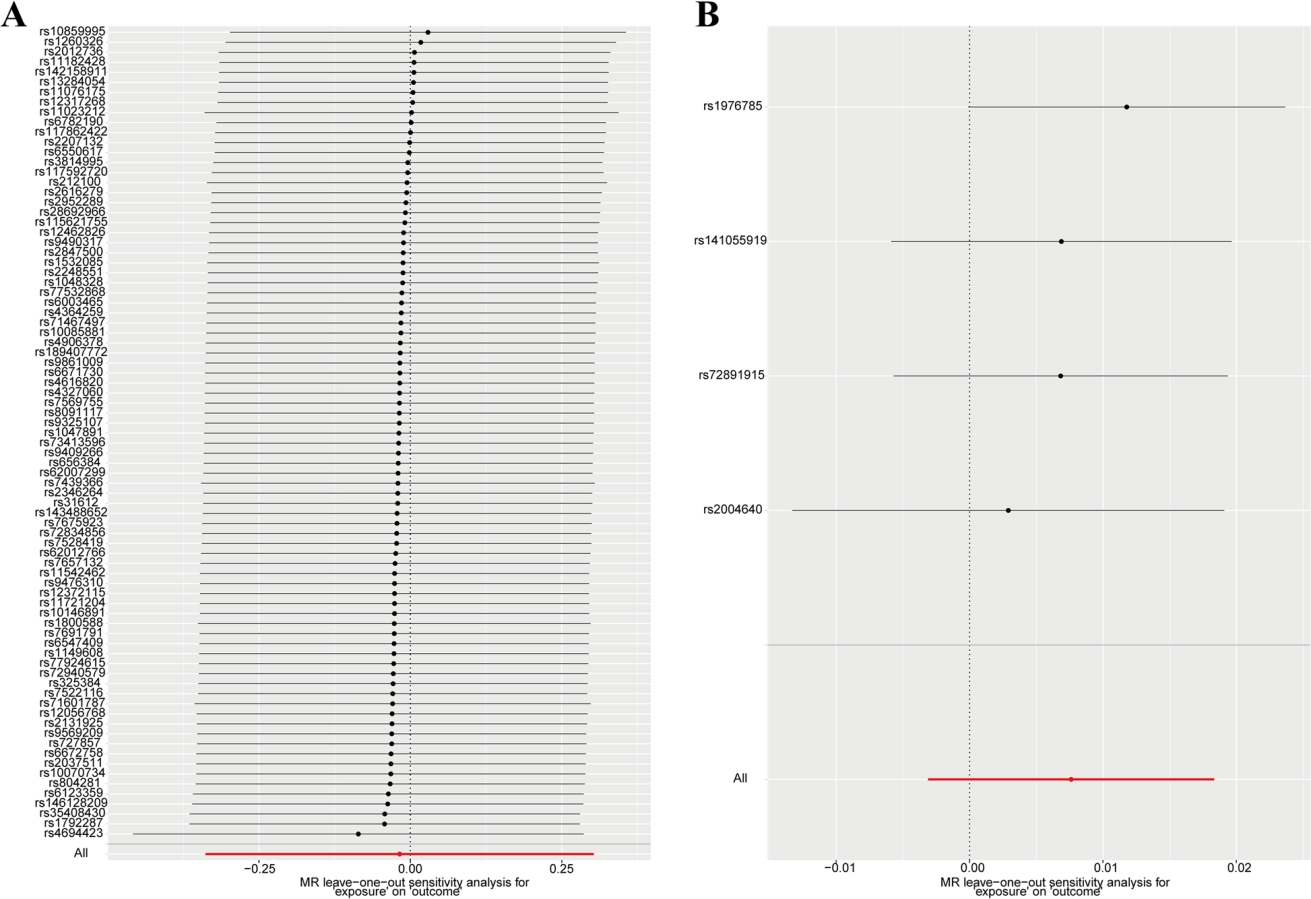
Leave-one-out sensitivity analysis of the effect of serum 25(OH)D levels on SS (**A**) and the effect of SS on serum 25(OH)D levels (**B**)

Our heterogeneity analysis showed that there was no heterogeneity between the data, as Cochran’s *Q* test was not statistically significant (*Q* = 71.3697, *P* = 0.7439). Moreover, in the MR-Egger test and the MR-PRESSO global test, no directional pleiotropy bias was evident (Tables 2 and 3). In addition, the leave-one-out analysis did not demonstrate any SNP outliers, which suggests that our results were stable (see Fig. 4).

## Discussion

To our knowledge, this is the first bi-directional MR design to assess the causal effects between serum vitamin D and SS. However, our data provided evidence supporting no association between vitamin D and SS using the MR approach.

Our MR analysis contradicts some of the existing observational studies that have proposed a correlation between serum vitamin D levels and SS. In a cohort study of 107 SS patients (97 female and 10 male) and 74 healthy controls (64 female and 10 male), Erten et al. found that vitamin D deficiency was frequent in patients with SS, and female SS patients had the risk of vitamin D deficiency [12]. Moreover, the latest systematic review and meta-analysis revealed that SS patients have lower serum vitamin D levels than controls [standardized mean difference (SMD) =  − 0.297; 95% CI: − 0.585 to − 0.01; *P* < 0.05] [14]. Although the correlation between serum vitamin D levels and SS was shown in the above studies, our MR study did not support a bi-directional causal association. This is consistent with the results of several other observational studies. Szodoray et al. found no statistical difference in serum vitamin D between 25 SS patients and 15 healthy individuals in a cross-sectional study [41]. In another cross-sectional survey, Agmon-Levin et al. similarly revealed no serological differences between 176 SS patients and 163 healthy individuals regarding vitamin D [13]. A possible explanation for the existence of such inconsistent results is that the previously observed associations between serum vitamin D levels and SS are coincidental or thwarted by an unknown confounder.

Moreover, a causal link between serum vitamin D levels and SS, or the converse, cannot be established in observational studies because SS is a systemic autoimmune disease accompanied by many complications, such as arthritis, rashes, pulmonary disease, renal or hepatic manifestations, central nervous system (CNS) involvement, and polyneuropathy [42]. Also, it is worth noting that vitamin D is a powerful immune modulator and that inflammation mutually regulates vitamin D metabolism [43, 44]. Therefore, we suspected the possibility that some inflammatory pathways would be shared between altered serum vitamin D levels and SS, leading to the conclusion in some observational studies that there is a correlation between the two phenotypes. Notably, the role of vitamin D has also been inconsistent in studies of its association with other autoimmune diseases. These include compulsory spondylitis [45], systemic lupus erythematosus [46], rheumatoid arthritis [47], and others. In sum, the underlying mechanism behind the relationship between vitamin D and SS is complex and worthy of further investigation. And due to the difficulty in observational epidemiological studies to eliminate the bias such as the reverse causal association of confounding factors, there are some limitations in etiological interpretation [48, 49].

In this study, the MR analysis satisfied three assumptions. For assumption I, the IVs are closely related to exposure. Eighty-one and four SNPs were selected in the GWAS, which was closely associated with vitamin D and SS, respectively, which verified assumption I. For assumption II, the IVs are independent of any potential confounding factor. LD between SNPs was assessed and screened, and we found that no SNPs were in LD with each other at an *r*^2^ > 0.05. For assumption III, the IVs only affect outcome via the exposure. The heterogeneity and sensitivity analyses have been conducted to detect and remove any potential pleiotropy, reassuring that our MR estimates are robust and reliable, with no perceptible bias from other sources of pleiotropy.

The strength of this study is the use of MR methods to minimize residual confounding and reverse causality in traditional observational studies. However, there are still some limitations that cannot be addressed at present. First, there should be no weak instrumental variable bias in this study because the *F*-statistics were all > 10; however, the low power may be caused by the low number of SNPs used as IVs. Meanwhile, this generated an impetus to perform MR studies on larger sample size populations. Second, the data obtained were GWAS summary data without specific personal information to the extent that subgroup analysis could not be performed. Third, because the population of European ancestry was used, we should be cautious about generalizing the findings to other populations. Meanwhile, the study needs to be repeated in future studies as the GWAS database continues to be improved and supplemented with sample size, sample information, etc.

## Conclusions

In neither direction did we find causal evidence in support of a causal association between serum vitamin D levels and SS risk. However, multi-center, large-scale GWAS cohort studies are in development and may extend the IVs in this study. The association between vitamin D levels and SS needs to be re-evaluated in future studies.

## Supplementary Information

**Additional file 1:** **Supplementary Table 1.** Summary of genetic variants (*n*=81) used to estimate the effect of serum vitamin D on SS in MR analyses.** Supplementary Table 2.** Summary of genetic variants (*n* = 4) used to estimate the effect of SS on serum vitamin D in MR analyses.

## Data Availability

The datasets used and/or analyzed during the current study are available from the corresponding author on reasonable request.

## Abbreviations

SS: Sjogren’s syndrome
MR: Mendelian randomization
GWAS: Genome-wide association studies
IVW: Inverse-variance weighted
25(OH)D: 25-Hydroxyvitamin D
IV: Instrumental variable
SNP: Single nucleotide polymorphism
RCT: Randomized controlled trial
LD: Linkage disequilibrium
EAF: Effect allele frequency
CI: Confidence interval
MR-PRESSO: MR-Pleiotropy Residual Sum and Outlier methods
OR: Odds ratio
SMD: Standardized mean difference
CNS: Central nervous system
