# Supplementary material for: Serum vitamin D levels and Sjogren’s syndrome: bi-directional Mendelian randomization analysis
Source: Arthritis Res Ther. 2023 May 15;25:79. doi: 10.1186/s13075-023-03062-2 (PMC10184420; doi:10.1186/s13075-023-03062-2)
Supplement: Supplementary file 1 — Additional file 1: Supplementary Table 1. Summary of genetic variants (n=81) used to estimate the effect of serum vitamin D on SS in MR analyses. Supplementary Table 2. Summary of genetic variants (n = 4) used to estimate the effect of SS on serum vitamin D in MR analyses. [file 13075_2023_3062_MOESM1_ESM.docx]

Supplementary Material

**Supplementary Table 1. Summary of genetic variants (n=81) used to estimate the effect of serum vitamin D on SS in MR analyses.**

| SNP | Chr | Pos (GRCh37) | Effect allele | Other allele | EAF | Beta | Se | P | R^2^ | F |
| --- | --- | --- | --- | --- | --- | --- | --- | --- | --- | --- |
| rs2131925 | 1 | 63025942 | G | T | 0.356375 | 0.02294 | 0.002085 | 3.61E-28 | 0.000241 | 100.8322 |
| rs35408430 | 1 | 17560195 | C | T | 0.657806 | 0.021495 | 0.0021 | 1.36E-24 | 0.000208 | 86.87665 |
| rs6671730 | 1 | 2339139 | G | A | 0.565714 | 0.014788 | 0.002011 | 1.92E-13 | 0.000107 | 44.87515 |
| rs6672758 | 1 | 230303512 | C | T | 0.199128 | -0.01759 | 0.002509 | 2.40E-12 | 0.000099 | 41.21332 |
| rs7522116 | 1 | 41835685 | C | T | 0.433767 | 0.013464 | 0.002025 | 2.97E-11 | 0.000089 | 37.18829 |
| rs7528419 | 1 | 109817192 | A | G | 0.775329 | -0.01974 | 0.002387 | 1.35E-16 | 0.000136 | 56.69619 |
| rs1047891 | 2 | 211540507 | C | A | 0.684179 | 0.015214 | 0.00214 | 1.18E-12 | 0.000100 | 41.77425 |
| rs1260326 | 2 | 27730940 | T | C | 0.393435 | -0.02061 | 0.002036 | 4.42E-24 | 0.000203 | 84.67632 |
| rs2012736 | 2 | 234622379 | C | A | 0.919186 | 0.048307 | 0.003666 | 1.16E-39 | 0.000347 | 144.8199 |
| rs6547409 | 2 | 21190209 | C | T | 0.950217 | -0.02612 | 0.004594 | 1.30E-08 | 0.000065 | 26.9554 |
| rs727857 | 2 | 58981967 | G | A | 0.388511 | 0.014018 | 0.002062 | 1.05E-11 | 0.000093 | 38.99171 |
| rs7569755 | 2 | 118648261 | G | A | 0.70942 | -0.01425 | 0.002212 | 1.18E-10 | 0.000084 | 34.96253 |
| rs11721204 | 3 | 49982765 | C | T | 0.561926 | -0.01364 | 0.002011 | 1.17E-11 | 0.000092 | 38.25276 |
| rs6550617 | 3 | 18777836 | A | G | 0.281502 | 0.012425 | 0.00222 | 2.19E-08 | 0.000062 | 26.07922 |
| rs6782190 | 3 | 85639672 | G | A | 0.352488 | 0.017216 | 0.002084 | 1.45E-16 | 0.000135 | 56.5045 |
| rs9861009 | 3 | 141654685 | T | C | 0.272485 | -0.01402 | 0.002253 | 4.86E-10 | 0.000078 | 32.54483 |
| rs11732896 | 4 | 88287993 | G | A | 0.701209 | 0.016005 | 0.002173 | 1.79E-13 | 0.000107 | 44.82709 |
| rs189407772 | 4 | 100146674 | A | G | 0.977306 | -0.0528 | 0.00672 | 3.90E-15 | 0.000124 | 51.6453 |
| rs4364259 | 4 | 15892159 | G | A | 0.797852 | -0.01591 | 0.002506 | 2.16E-10 | 0.000082 | 34.0985 |
| rs4616820 | 4 | 57745481 | C | T | 0.535046 | 0.012286 | 0.002018 | 1.13E-09 | 0.000075 | 31.36334 |
| rs4694423 | 4 | 72554159 | C | A | 0.583561 | 0.100742 | 0.002022 | 1.00E-200 | 0.004933 | 2070.017 |
| rs71601787 | 4 | 72866015 | G | A | 0.673283 | -0.04226 | 0.002142 | 1.22E-86 | 0.000786 | 328.3502 |
| rs7439366 | 4 | 69964338 | T | C | 0.544441 | 0.032229 | 0.001997 | 1.41E-58 | 0.000515 | 215.2685 |
| rs7657132 | 4 | 73416601 | A | G | 0.683314 | 0.014251 | 0.00217 | 5.14E-11 | 0.000088 | 36.70673 |
| rs7675923 | 4 | 72345872 | A | G | 0.15599 | -0.02562 | 0.002759 | 1.63E-20 | 0.000173 | 72.18472 |
| rs10070734 | 5 | 87940026 | T | C | 0.290469 | -0.01321 | 0.002198 | 1.82E-09 | 0.000072 | 30.03832 |
| rs31612 | 5 | 108996643 | T | C | 0.825562 | 0.014528 | 0.002649 | 4.15E-08 | 0.000061 | 25.38613 |
| rs9325107 | 5 | 148016857 | G | T | 0.558134 | -0.0112 | 0.002027 | 3.23E-08 | 0.000062 | 25.83804 |
| rs2248551 | 6 | 131924689 | G | A | 0.834778 | 0.023362 | 0.002682 | 3.04E-18 | 0.000151 | 62.87701 |
| rs72834856 | 6 | 22801858 | T | G | 0.927936 | 0.024987 | 0.003851 | 8.67E-11 | 0.000084 | 34.87133 |
| rs9476310 | 6 | 57767576 | C | T | 0.488637 | -0.01176 | 0.002001 | 4.21E-09 | 0.000069 | 28.8621 |
| rs9490317 | 6 | 121859499 | T | C | 0.554106 | -0.01105 | 0.002012 | 3.95E-08 | 0.000060 | 25.19665 |
| rs10085881 | 7 | 21577960 | T | C | 0.717815 | 0.014558 | 0.002238 | 7.83E-11 | 0.000086 | 35.85542 |
| rs2346264 | 7 | 133536351 | A | C | 0.217315 | 0.013883 | 0.002436 | 1.20E-08 | 0.000066 | 27.38038 |
| rs12056768 | 8 | 116988527 | T | G | 0.417091 | 0.023403 | 0.002024 | 6.44E-31 | 0.000266 | 111.2392 |
| rs28692966 | 8 | 25892919 | G | A | 0.747064 | -0.01483 | 0.0023 | 1.13E-10 | 0.000083 | 34.70999 |
| rs804281 | 8 | 11611865 | A | G | 0.416395 | -0.0133 | 0.002021 | 4.72E-11 | 0.000086 | 35.90317 |
| rs13284054 | 9 | 107669073 | T | C | 0.882273 | -0.01757 | 0.003134 | 2.07E-08 | 0.000064 | 26.78046 |
| rs9409266 | 9 | 125745042 | G | A | 0.138899 | 0.019633 | 0.002881 | 9.45E-12 | 0.000092 | 38.50651 |
| rs77532868 | 10 | 88081438 | C | T | 0.945958 | -0.02657 | 0.004401 | 1.56E-09 | 0.000072 | 30.14292 |
| rs11023212 | 11 | 14431709 | G | A | 0.65596 | 0.071521 | 0.002119 | 1.00E-200 | 0.002309 | 966.3288 |
| rs1149608 | 11 | 76494928 | C | T | 0.824922 | -0.02132 | 0.002635 | 6.00E-16 | 0.000131 | 54.83319 |
| rs117592720 | 11 | 14980137 | T | C | 0.973916 | -0.06969 | 0.006278 | 1.26E-28 | 0.000247 | 103.065 |
| rs117862422 | 11 | 13210063 | T | C | 0.986407 | 0.054735 | 0.008635 | 2.32E-10 | 0.000080 | 33.5509 |
| rs143488652 | 11 | 13508611 | A | G | 0.986616 | 0.063888 | 0.008686 | 1.91E-13 | 0.000108 | 45.01809 |
| rs146128209 | 11 | 14683683 | A | G | 0.92915 | 0.057527 | 0.003891 | 1.86E-49 | 0.000436 | 182.0229 |
| rs1792287 | 11 | 71057911 | A | G | 0.735113 | -0.02193 | 0.002266 | 3.78E-22 | 0.000187 | 78.22417 |
| rs2847500 | 11 | 120114421 | G | A | 0.876497 | 0.021925 | 0.003028 | 4.42E-13 | 0.000104 | 43.46303 |
| rs71467497 | 11 | 70471414 | T | C | 0.971397 | 0.034605 | 0.006008 | 8.40E-09 | 0.000067 | 27.78962 |
| rs72940579 | 11 | 66182450 | C | T | 0.661911 | -0.01236 | 0.002144 | 8.23E-09 | 0.000068 | 28.55386 |
| rs10859995 | 12 | 96375682 | T | C | 0.417366 | 0.040347 | 0.002021 | 1.06E-88 | 0.000792 | 330.8621 |
| rs11182428 | 12 | 38526387 | T | C | 0.480005 | 0.012535 | 0.001994 | 3.23E-10 | 0.000078 | 32.75633 |
| rs12317268 | 12 | 21352541 | A | G | 0.848996 | 0.020897 | 0.002785 | 6.19E-14 | 0.000112 | 46.7604 |
| rs12372115 | 12 | 97982701 | G | T | 0.929281 | 0.021795 | 0.003879 | 1.93E-08 | 0.000062 | 26.07304 |
| rs73413596 | 12 | 111582630 | T | C | 0.926146 | -0.0217 | 0.003826 | 1.41E-08 | 0.000064 | 26.90101 |
| rs9569209 | 13 | 55707745 | C | T | 0.714424 | 0.01265 | 0.002203 | 9.35E-09 | 0.000065 | 27.26809 |
| rs10146891 | 14 | 29719646 | C | T | 0.643603 | -0.01282 | 0.002087 | 8.04E-10 | 0.000075 | 31.4868 |
| rs4906378 | 14 | 104283445 | C | T | 0.661154 | 0.012827 | 0.002109 | 1.19E-09 | 0.000074 | 30.78611 |
| rs1532085 | 15 | 58683366 | A | G | 0.385165 | -0.02619 | 0.002046 | 1.55E-37 | 0.000325 | 135.7016 |
| rs1800588 | 15 | 58723675 | C | T | 0.784797 | 0.032922 | 0.002422 | 4.38E-42 | 0.000366 | 152.9342 |
| rs325384 | 15 | 100229761 | C | T | 0.715795 | 0.014173 | 0.002218 | 1.66E-10 | 0.000082 | 34.13085 |
| rs62007299 | 15 | 77711719 | G | A | 0.287463 | 0.013341 | 0.0022 | 1.32E-09 | 0.000073 | 30.44847 |
| rs62012766 | 15 | 63852834 | T | C | 0.84289 | 0.017172 | 0.002737 | 3.53E-10 | 0.000078 | 32.61505 |
| rs11076175 | 16 | 57006378 | A | G | 0.821642 | -0.02305 | 0.002607 | 9.48E-19 | 0.000156 | 65.03578 |
| rs11542462 | 16 | 82033810 | G | A | 0.865656 | 0.023334 | 0.002918 | 1.27E-15 | 0.000127 | 52.88891 |
| rs4327060 | 16 | 72807438 | C | T | 0.945604 | 0.024359 | 0.004392 | 2.92E-08 | 0.000061 | 25.49114 |
| rs77924615 | 16 | 20392332 | G | A | 0.806515 | 0.016632 | 0.002552 | 7.11E-11 | 0.000086 | 36.05403 |
| rs2952289 | 17 | 66464414 | C | T | 0.201968 | -0.01772 | 0.002492 | 1.18E-12 | 0.000101 | 42.27094 |
| rs2037511 | 18 | 61366207 | G | A | 0.833993 | -0.01812 | 0.00268 | 1.35E-11 | 0.000091 | 37.96752 |
| rs656384 | 18 | 57906500 | G | A | 0.733996 | 0.012832 | 0.002257 | 1.31E-08 | 0.000064 | 26.85135 |
| rs8091117 | 18 | 28919794 | C | A | 0.934702 | 0.026363 | 0.004028 | 5.98E-11 | 0.000085 | 35.42967 |
| rs1048328 | 19 | 51527364 | G | A | 0.919753 | -0.02844 | 0.003666 | 8.66E-15 | 0.000119 | 49.86303 |
| rs12462826 | 19 | 11955767 | G | A | 0.631278 | 0.012375 | 0.00208 | 2.70E-09 | 0.000071 | 29.77205 |
| rs142158911 | 19 | 11190534 | G | A | 0.885392 | -0.02553 | 0.003146 | 4.78E-16 | 0.000132 | 55.24299 |
| rs212100 | 19 | 48376995 | T | C | 0.164001 | 0.066152 | 0.00269 | 1.60E-133 | 0.001200 | 501.6806 |
| rs3814995 | 19 | 36342212 | C | T | 0.688405 | 0.012558 | 0.00215 | 5.18E-09 | 0.000068 | 28.25351 |
| rs2207132 | 20 | 39142516 | G | A | 0.96711 | 0.034596 | 0.005578 | 5.56E-10 | 0.000076 | 31.79744 |
| rs2616279 | 20 | 52743897 | C | T | 0.848239 | 0.022648 | 0.002782 | 3.89E-16 | 0.000132 | 55.15223 |
| rs6123359 | 20 | 52714706 | A | G | 0.897775 | -0.03418 | 0.003314 | 6.10E-25 | 0.000214 | 89.56318 |
| rs115621755 | 22 | 50853134 | C | T | 0.67288 | 0.012431 | 0.002123 | 4.76E-09 | 0.000068 | 28.40887 |
| rs6003465 | 22 | 23365501 | T | C | 0.668006 | 0.011962 | 0.002123 | 1.77E-08 | 0.000063 | 26.50412 |

Note: SNP, single nucleotide polymorphism; Chr, chromosome; EAF, effect allele frequency; SE, standard error.

**Supplementary Table 2. Summary of genetic variants (n = 4) used to estimate the effect of SS on serum vitamin D in MR analyses.**

| SNP | Chr | Pos (GRCh37) | Effect allele | Other allele | EAF | Beta | Se | P | R^2^ | F |
| --- | --- | --- | --- | --- | --- | --- | --- | --- | --- | --- |
| rs141055919 | 6 | 33895564 | C | T | 0.062441 | 0.352748 | 0.059201 | 2.55E-09 | 0.014569 | 6161.439 |
| rs72891915 | 6 | 33476200 | A | G | 0.040002 | 0.469082 | 0.070813 | 3.49E-11 | 0.016900 | 7164.115 |
| rs2004640 | 7 | 128578301 | G | T | 0.477583 | -0.27222 | 0.031776 | 1.06E-17 | 0.036977 | 16002.23 |
| rs1976785 | 11 | 36894806 | T | C | 0.377844 | -0.17117 | 0.033382 | 2.93E-07 | 0.013775 | 5821.059 |

Note: SNP, single nucleotide polymorphism; Chr, chromosome; EAF, effect allele frequency; SE, standard error.
